# *Nocturnin* Expression Is Induced by Fasting in the White Adipose Tissue of Restricted Fed Mice

**DOI:** 10.1371/journal.pone.0017051

**Published:** 2011-02-10

**Authors:** Misty R. Gilbert, Nicholas Douris, Siripong Tongjai, Carla B. Green

**Affiliations:** Department of Biology, University of Virginia, Charlottesville, Virginia, United States of America; Vanderbilt University, United States of America

## Abstract

The relationship between circadian clocks and metabolism is intimate and complex and a number of recent studies have begun to reveal previously unknown effects of food and its temporal availability on the clock and the rhythmic transcriptome of peripheral tissues. *Nocturnin*, a circadian deadenylase, is expressed rhythmically in a wide variety of tissues, but we report here that *Nocturnin* expression is arrhythmic in epididymal white adipose tissue (eWAT) of mice housed in 12∶12 LD with *ad libitum* access to food. However, *Nocturnin* expression becomes rhythmic in eWAT of mice placed on restricted feeding. We show here that *Nocturnin's* rhythmic expression pattern is not dependent upon feeding, nor is it acutely induced by feeding in the liver or eWAT of *ad libitum* fed mice. However, *Nocturnin* is acutely induced by the absence of the expected meal in eWAT of restricted fed mice. A rise in cAMP levels also induces *Nocturnin* expression, suggesting that *Nocturnin's* induction in eWAT by fasting is likely mediated through the same pathways that activate lipolysis. Therefore, this suggests that *Nocturnin* plays a role in linking nutrient sensing by the circadian clock to lipid mobilization in the adipocytes.

## Introduction

Circadian clocks orchestrate a variety of physiological and molecular rhythms that allow organisms to anticipate and respond to the daily changes that occur in the environment such as light, temperature, and feeding/fasting cycles. Restricted feeding studies have revealed that food can act as a zeitgeber (an entraining signal or ‘timegiver’), and have demonstrated that peripheral oscillators can be uncoupled from the master pacemaker located in the suprachiasmatic nucleus (SCN) [1], [2], [3]. Besides the entrainment of the circadian clock by food, there is a growing body of evidence that demonstrates that there is cross-talk between circadian clocks and metabolism and that signals from each feedback onto the other [4]. For example, mutations or deficiencies in core clock genes such as *Clock* or *Bmal1* result in obesity, lipodystrophy and/or glucose homeostasis abnormalities [5], [6], [7], [8], [9], and nuclear receptors central to regulating metabolic processes, such as PPARα and PGC-1α, affect the circadian clock [10], [11].

The connection between circadian biology and metabolism is not limited to the core clock machinery, but extends to genes downstream of the clock as well. For example, loss of the rhythmically expressed deadenylase *Nocturnin* (*Ccrn4l*) results in metabolic abnormalities [12], [13], [14]. *Nocturnin* expression peaks in the early night in a variety of tissues such as the liver, the kidney, the spleen, and the retina [13]. *Nocturnin* deficient mice show no abnormalities in circadian behavior nor in the rhythmic expression of core clock genes in the liver, showing that *Nocturnin* is not part of the core circadian mechanism, but rather contributes to the rhythmic output of the clock [12]. *Nocturnin* ^−/−^ mice are resistant to diet-induced obesity and hepatic steatosis, and show dampened rhythms or decreased expression levels of genes related to lipid metabolism in the liver, such as *Pparγ* and *Scd1*, respectively [12].

It has also been demonstrated that *Nocturnin* is the only known mammalian deadenylase that responds acutely to extracellular stimuli: It is induced in NIH-3T3 fibroblasts by serum and by the phorbol ester 12-O-tetradecanoylphorbol-13-acetate (TPA), and it is also acutely induced by insulin in 3T3-L1 preadipocytes [14], [15]. However, the *in vivo* significance of this acute responsiveness has not been clear.

In mice that conditionally over-express REV-ERBα in the liver, thus rendering the circadian clock nonfunctional in this tissue, *Nocturnin* mRNA levels remain rhythmic with a peak expression time occurring in the early night [16]. This finding suggests that *Nocturnin's* rhythmicity in the liver can be driven by systemic cues. However, it is unclear whether *Nocturnin's* expression pattern in the liver, and perhaps in other tissues, is due to an acute induction by extracellular stimuli, or whether it is the result of an entrained mechanism independent of the oscillation of the local liver clock.

White adipose tissue plays a pivotal role in energy homeostasis by storing energy in the form of triglycerides after a meal, and undergoing lipolysis during times of fasting. It also functions as an endocrine organ secreting a variety of adipokines that have systemic affects on appetite and glucose homeostasis [17], [18], [19]. Due to *Nocturnin's* role in metabolism, its inducibility by extracellular stimuli *in vitro*, and its capability to be driven by systemic cues in the liver, we set out to examine *Nocturnin's* expression pattern in white adipose tissue and to investigate whether *Nocturnin* can be acutely induced by feeding or fasting cues in the liver and the white adipose tissue, two tissues central to metabolic homeostasis. We report that *Nocturnin's* expression pattern is not rhythmic in white adipose tissue in *ad libitum* feeding conditions, but is rhythmic in white adipose tissue of mice subjected to temporal, not caloric, restriction of food availability. Also, we show that *Nocturnin* is acutely induced by fasting in the white adipose tissue of restricted-fed mice.

## Results

### *Nocturnin* expression is not dependent upon or induced by feeding

**Figure 1:**
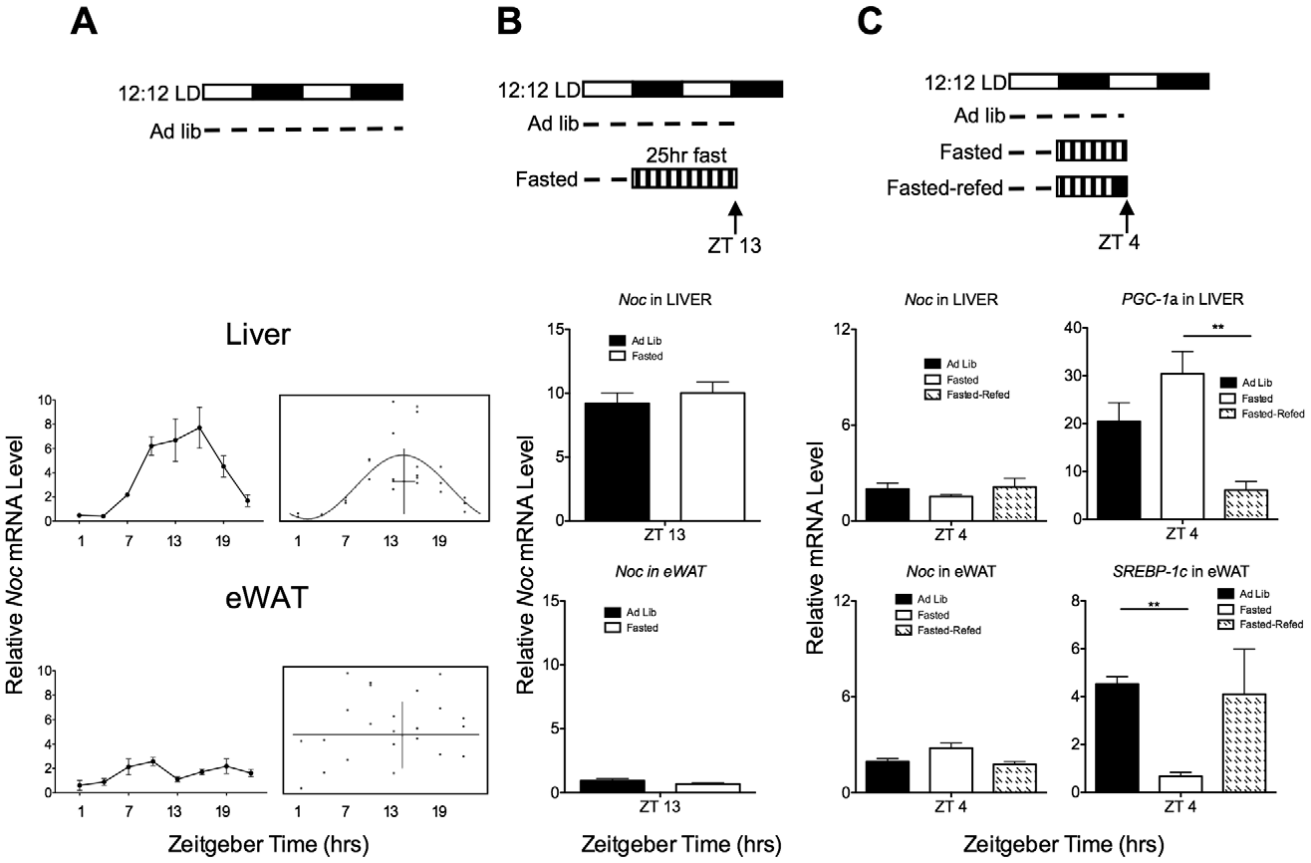
*Nocturnin* expression is not dependent upon or induced by feeding. (**A**) *Nocturnin* expression is not dependent upon feeding. The top panel is a schematic depicting the experimental setup. Open bars represent lights on, and the closed bars represent lights off. The dotted line depicts food availability. Graphs (left) represent the mean relative mRNA level (±SEM) for each time point in liver (top) and eWAT (bottom), n = 3–6. CircWave analysis (right panels of A) shows the corresponding statistical analyses for rhythmicity. In the CircWave graphs, each dot represents the normalized relative mRNA level from the eWAT of an individual mouse. The curve represents the best-fit fourier curve, with the center of gravity (phase) represented by the vertical bar intersecting with the horizontal bar which represents the mean of the entire data set (liver; p≤0.002). Data set for which a fourier curve could not be fitted (eWAT) is indicated by ‡. (**B**) *Nocturnin* expression is not induced by fasting. Schematic of fasting experiment (top panel) as described in (A). The bar with vertical stripes represents the fasting period. Graphs represent the mean (±SEM) for each group, n = 4. (**C**) *Nocturnin* expression is not induced by feeding after a fast. Schematic representation of experiment is as described in (A) and (B). The solid black bar in the “refed” condition represents the time period in which food was made available after the fast. Gene expression analysis of the liver (middle panel) and eWAT (bottom panel) collected at ZT4 shows that *Nocturnin* is not induced by feeding after a fast, though the expected changes in expression patterns of *Srebp-1c* and *Pgc-1α* were observed (see text). Data represent the mean (±SEM), n = 3 for each group.

The fact that *Nocturnin* was acutely induced by stimuli such as TPA and insulin *in vitro* raised the possibility that *Nocturnin* may be acutely induced in response to feeding [14], [15]. *Nocturnin* expression peaks in the early night in the livers of *ad libitum* fed mice housed in 12∶12 light:dark (LD) conditions (Figure 1A; [12], [13]). However, quantitative real-time PCR analysis of the epididymal white adipose tissue (eWAT) from *ad libitum* fed mice housed in 12∶12 light:dark revealed that *Nocturnin* is expressed at low levels, and is statistically arrhythmic (Figure 1A, bottom panel). *Nocturnin* expression is arrhythmic despite rhythmic core clock gene expression in eWAT [20], [21]. The observation that *Nocturnin's* expression pattern can be driven by systemic cues in the liver with a peak expression in the early night [16] led us to hypothesize that this expression pattern could be the result of an acute induction of *Nocturnin* caused by the large bout of feeding which begins at the onset of darkness [22]. To test this, mice were habituated to a 12∶12 LD cycle and then fasted for 25 hours, beginning at ZT12 (ZT refers to “zeitgeber time” in hours, where ZT0 is light onset and ZT12 is light offset) and ending at ZT13 the next day (Figure 1B, top panel). Contrary to our hypothesis, we found that *Nocturnin* expression both in the liver and in the white adipose tissue was not dependent upon the presence of food (Figure 1B, bottom panel).

Though *Nocturnin* peak expression in the early night was not affected by the absence of food, it remained to be determined if *Nocturnin* could be acutely induced by feeding if the feeding occurred at the nadir of its regular expression pattern. To test this, a group of mice were fasted overnight, beginning at ZT12, and were re-fed at ZT2 (Figure 1C, top panel). Gene expression analysis of the liver and of the white adipose tissue, taken two hours after feeding began, revealed that *Nocturnin* expression levels remained unchanged from the *ad libitum* and fasted controls, and were not induced by feeding (Figure 1C). As previously reported, *Srebp-1c* expression was down-regulated in eWAT and *Pgc-1α* expression was up-regulated in liver in response to the fast (Figure 1C; [23]). Therefore, these experiments demonstrate that *Nocturnin* expression is not dependent upon, nor induced, by feeding.

### Restricted feeding induces the rhythmicity of *Nocturnin* in white adipose tissue

**Figure 2:**
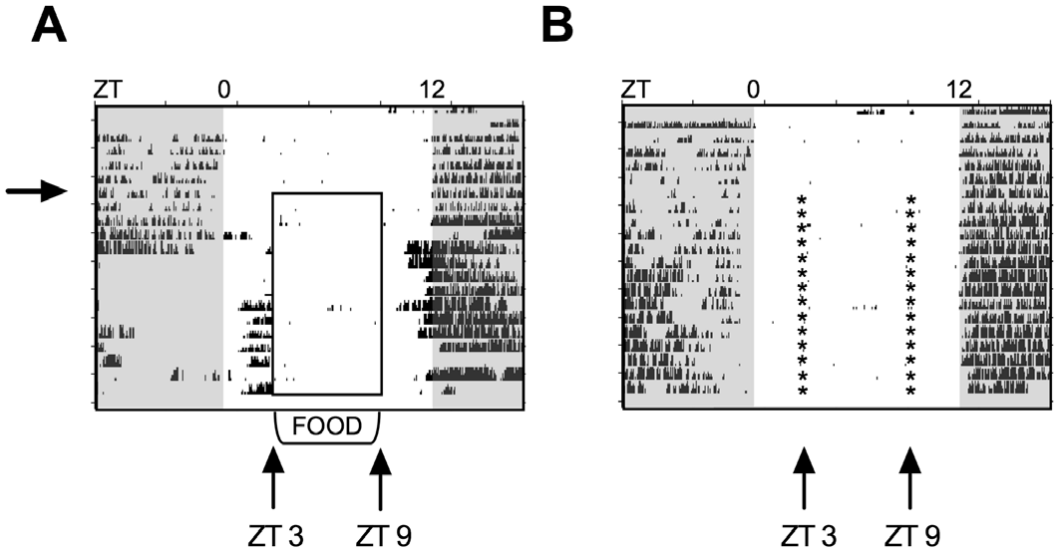
Wheel-running actograms of restricted fed or *ad libitum* fed C57BL/6 mice. (**A**) Restricted fed mice. Each horizontal line of the actogram represents a twenty-four hour period, and each tick mark on the graph represents one running-wheel revolution. Lights off are indicated by the shaded areas of the actograms. Mice were habituated to a 12∶12 LD cycle for 7 days with *ad libitum* access to food. At ZT9 on Day 7 (marked by the arrow), food was removed from the cages. At ZT3, a small bowl containing food was placed in the bottom of the cage. At ZT9 the food was removed. This was repeated daily until the conclusion of the experiment on Day 21. Food availability is marked by the open box. This actogram is a representative example from an individual mouse. (**B**) *Ad libitum* fed controls. Actogram is as described in (A). Mice were habituated to a 12∶12 LD cycle for 7 days. On day 8, small bowls of food were placed into the cages, and food was moved in and out of the bowl at ZT3 and ZT9, respectively. Controls were maintained on *ad libitum* feeding throughout the study, but on day 8 small bowls of food were placed into the cages, and food was moved in and out of the cage in the same manner as the restricted fed mice. Times food bowls were moved in and out are represented by *.

**Figure 3:**
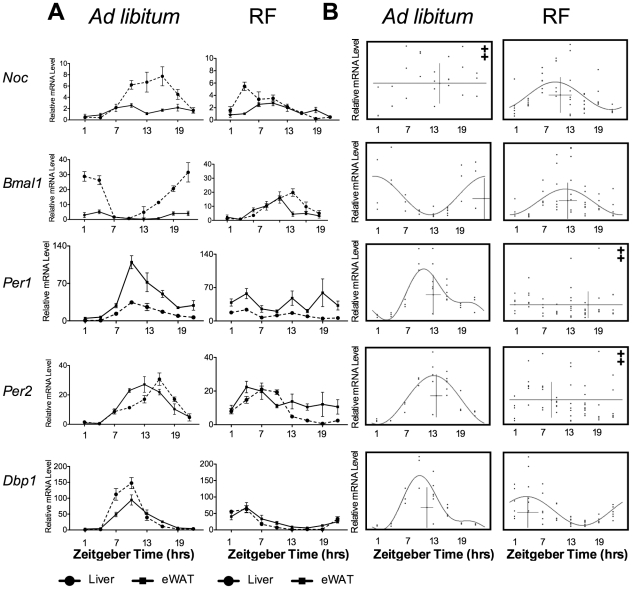
Restricted feeding results in the rhythmic expression of *Nocturnin* in the epididymal white adipose tissue. (**A**) Gene expression analysis conducted on the liver (dashed line) and on the eWAT tissue (solid line) in *ad libitum* fed mice (left panel) and restricted fed mice (RF, right panel). Data represent the mean (± SEM) of a group for each time point (n = 3–6 per timepoint for the *ad libitum* condition, and n = 5–9 for the restricted fed condition). *Ad libitum* expression profiles of *Nocturnin* shown in 3A are the same data as shown in Figure 1A, and is regraphed here for convenient comparison. (**B**) CircWave analysis of eWAT gene expression data from (A) from *ad libitum* (left panel) and restricted fed mice (RF, right panel). The graphs are as described in Figure 1A. For all gene expression values, except those graphs marked with ‡, p-values <0.002. ‡ marks the data for which no curve could be fit. Note that the *Nocturnin ad libitum* data from liver and eWAT in this figure are the same data as shown in Figure 1A and are shown again here for direct comparison.

To investigate the effect that restricted feeding would have on the expression patterns of *Nocturnin*, mice were placed on a feeding paradigm in which food availability was temporally restricted to six hours during the middle of the day, from ZT3 to ZT9 (Figure 2A). Mice in the control group were mock restricted fed by disturbing the cages at ZT3 and ZT9, though the mice had *ad libitum* access to food during the entire study (Figure 2B). Restricted fed mice displayed food anticipatory wheel running activity within three or four days after the onset of food restriction, whereas the *ad libitum* fed group did not (Figure 2A,B). This indicates that food entrainment has occurred in the experimental group. Gene expression analysis of *ad libitum* fed mice revealed that *Nocturnin* expression, along with other core clock genes, was rhythmic in the liver, as previously reported (Figure 1A and 3A, left panel; [12], [13]).

We found that the effect of restricted feeding was tissue- and gene-dependent. When mice were placed on the restricted feeding paradigm, *Nocturnin's* expression pattern in the liver shifted from a peak in the early night to a peak at ZT4 (Figure 3A, right panel). Though *Nocturnin's* expression pattern was arrhythmic in the white adipose tissue of *ad libitum* fed mice, restricted feeding resulted in the rhythmic expression of *Nocturnin*, which displayed a peak at ZT10, in this tissue (Figure 3B, right panel). Also in the white adipose tissue, the expression patterns of *Bmal1* and *Dbp1* shifted to resemble the profiles seen in the liver (Figure 3A, right panel), while the *Per1* and *Per2* expression patterns were arrhythmic in restricted-fed mice, unlike the expression profile of these genes in the liver (Figure 3A, right panel and Figure 3B, right panel).

### *Nocturnin* expression is induced by the absence of the meal in the white adipose tissue of restricted-fed mice

**Figure 4:**
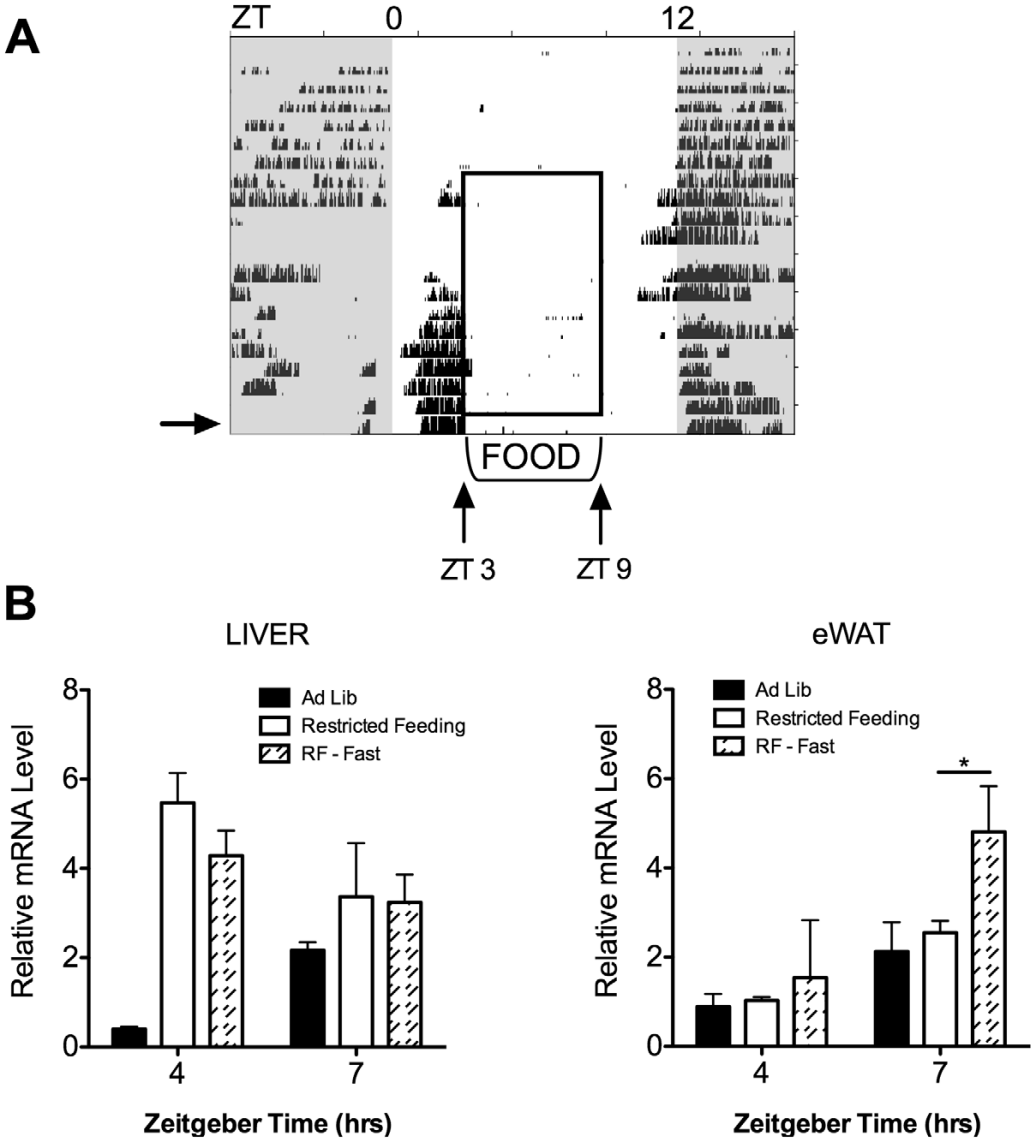
*Nocturnin* is induced by fasting in eWAT tissue of restricted fed mice. (**A**) Schematic representation of feeding paradigm and absence of meal on the thirteenth day of restricted feeding. Actogram is as described in Figure 2, and is a representative wheel running actogram of restricted fed mice. Mice were habituated to a 12∶12 LD cycle for seven days, restricted feeding began on Day 8. On the 13th day of restricted feeding (indicated by the arrow), mice were not given the expected meal at ZT3, the condition labeled as RF-Fast. (**B**) Gene expression analysis of the liver (left panel) and the epididymal white adipose tissue (eWAT, right panel) reveal that *Nocturnin* is induced by the absence of the meal in eWAT only. The data from the *ad libitum* control group and from the restricted fed group (RF) are the same as from Figure 2 and has been re-graphed here for comparison. Data represent the mean of the group (±SEM); for each time point, n = 3 for the *ad libitum* group, n = 6 for the restricted-fed group, and n = 4 for the RF-fasted group. *  =  t-test, p-value  = 0.019.

In restricted-fed mice, the peak expression of *Nocturnin* in liver and in eWAT occurs at ZT4 and ZT10, respectively. To test whether peak expression was dependent upon the presence of the expected meal in this paradigm, we placed animals on a restricted feeding schedule for 12 days and on the thirteenth day no meal was presented at ZT3 (Figure 4A). Tissues were harvested both at ZT4 and at ZT7. Gene expression analysis revealed that *Nocturnin* was unaffected by the absence of the meal in the liver (Figure 4B, left panel). However, in the white adipose tissue of restricted fed mice, *Nocturnin* expression was induced approximately two-fold at ZT7 in response to the fast (Figure 4B, right panel).

### *Nocturnin* expression is induced by a rise in cAMP levels in adipocytes

**Figure 5:**
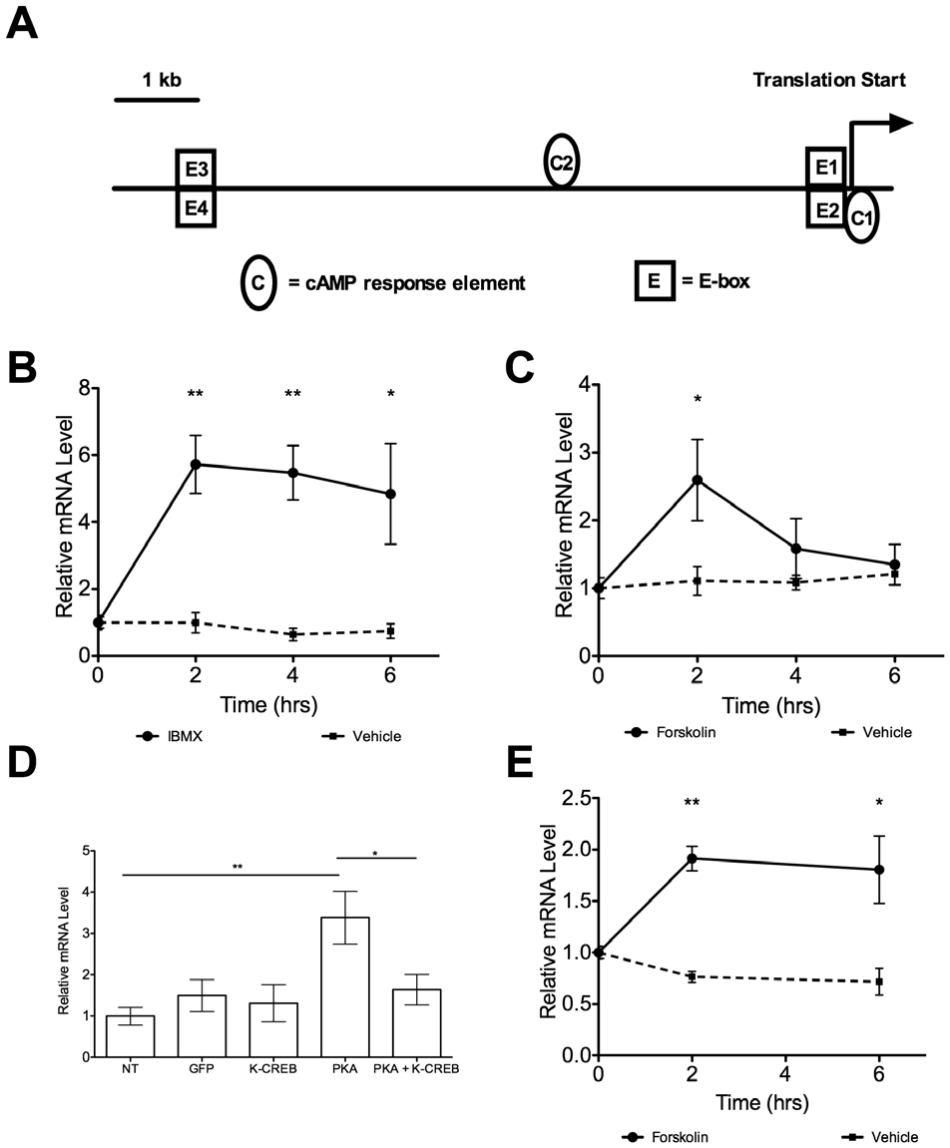
*Nocturnin* is induced by a rise in cAMP in NIH-3T3 fibroblasts and in 3T3-L1 adipocytes. (**A**) Schematic representation of highly conserved putative transcription factor binding sites in the 10 kb region upstream of *Nocturnin's* translation start site. C =  cAMP response element binding protein (CRE) sites. E =  E-box elements. Locations of the CRE sites are C1 −29 bp and C2+3,016 bp. (**B**) *Nocturnin* is induced approximately 4.5 fold by IBMX (solid line) in NIH3T3 fibroblasts. No induction of *Nocturnin* was observed in the vehicle treated cells (dashed line). Data represent mean (± SEM), normalized to *Cyclophilin B*. n = 4 for each time point. P-value determined by t-test assuming equal variances, two-way p-value reported. ** p-value <0.002; * p-value <0.03. (C) *Nocturnin* is induced approximately 2.5 fold by forskolin (solid line) in NIH3T3 fibroblasts. The dashed line represents data from vehicle treated cells. Data represent mean (± SEM), normalized to *Cyclophilin B*. n = 3 for each time point. P-value determined by t-test assuming equal variances, two-way p-value reported. * p-value <0.03. (D) Transient transfection assay of NIH3T3 fibroblasts showing *Nocturnin* is induced by the overexpression of PKA in a CREB-dependent manner. Data represent the mean (± SEM), n = 6 for each group. T-test assuming equal variances performed, p-value reported. **  =  p-value <0.01; *  =  p-value <0.04. NT =  non-transfected; GFP =  GFP transfected; K-CREB =  pCMV-KCREB (dominant negative) alone transfected; PKA =  pCMV-PKA transfected; PKA + K-CREB  =  pCMV-PKA and dominant negative pCMV-KCREB were cotransfected. (E) *Nocturnin* is induced approximately 2 fold by forskolin (solid line) in mature 3T3-L1 adipocytes. The dashed line represents data from vehicle treated cells. Data represent the mean (± SEM), n = 3. **  =  p-value 0.0009, and *  =  p-value 0.036. Data were normalized to *Cyclophilin B*.

During fasting, white adipose tissue undergoes lipolysis mediated by catecholamine stimulated beta-adrenergic receptors which causes a rise in cAMP levels, leading to the activation of protein kinase A (PKA) which results in the phosphorylation of hormone sensitive lipase (HSL), perilipin A, and cAMP response element binding protein (CREB), and ultimately the breakdown of triglyceride-storing lipid droplets [24], [25], [26], [27]. It has been shown in *Xenopus laevis* that *Nocturnin* rhythmicity is regulated by p-CREB, but the activation of *Nocturnin* by p-CREB has not been demonstrated in mice [28]. There are conserved putative CREs within the 500 bp region upstream of *Nocturnin's* first exon (Figure 5A), and *Nocturnin* is acutely induced by 3-isobutyl-1-methylxanthine (IBMX) and forskolin in NIH3T3 fibroblasts (Figure 5B,C). Overexpression of PKA in NIH-3T3 fibroblasts resulted in an increase of *Nocturnin* expression, whereas cells co-transfected with PKA and a dominant negative CREB construct failed to exhibit the increase in *Nocturnin* expression, demonstrating that mammalian *Nocturnin* mRNA expression is induced by CREB activation (Figure 5D). We next examined whether *Nocturnin* could be induced by a rise in cAMP levels in mature 3T3-L1 adipocytes. Treatment of these cells with forskolin also resulted in an acute induction of *Nocturnin* expression (Figure 5E) showing that *Nocturnin* is regulated by the signaling cascade which occurs during lipolysis in white adipose tissue.

## Discussion

*Nocturnin* ^−/−^ mice have no change in circadian locomotor activity rhythms or in the expression patterns of canonical clock genes in the liver suggesting that *Nocturnin* is an output of the circadian clock [12]. The observation that *Nocturnin* expression is not acutely induced by food nor by the absence of the expected meal in the livers of restricted fed mice suggests that the regulation of *Nocturnin* expression in the liver is likely the result of the local circadian clock, a food entrained oscillator, a systemic cue that is not dependent upon the daily presentation of food, or some combination thereof. Since *Nocturnin* shows robust rhythmicity in most tissues where it has been examined [13], our finding that *Nocturnin* is not strongly rhythmic in white adipose tissue under an *ad libitum* feeding paradigm was surprising. This finding suggests *Nocturnin* may be uncoupled from the regulation of the core clock machinery or influenced by other signals that are dominant to the clock in this situation.

A recent study demonstrated that the temporal pattern of feeding, as well as the availability of food, greatly affected the phase of the rhythmic expression of the transcriptome in the liver, and even is capable of partially restoring rhythmic gene expression patterns in the otherwise arrhythmic *Cry1* ^−/−^;*Cry2* ^−/−^ mice [22]. Interestingly, *Nocturnin* becomes rhythmically expressed in the white adipose tissue of restricted fed mice, indicating that its rhythmic expression pattern is driven by the temporal availability of food. *Nocturnin's* peak expression time in the white adipose tissue of restricted fed mice is six hours later than the peak expression time in the liver, implying that different mechanisms modulate this effect. It has been shown that peripheral tissues respond to food entrainment differently, as the phase of peak *Per1* expression between the lung and the liver differ, and the liver entrains to the feeding schedule faster than the lung. It was suggested that this difference could be due to the liver's important role in metabolism [2]. Our data contrasting the *Nocturnin* expression patterns in the liver and in the white adipose tissue of restricted fed mice suggest that even tissues directly involved in metabolic homeostasis have differing responses to food entrainment. Our data not only demonstrate a tissue-specific expression pattern of *Nocturnin*, but also demonstrate that the expression patterns of some core clock genes are tissue-specific.

In a restricted feeding paradigm, we have shown that *Per1* and *Per2* expression patterns become highly variable in eWAT and are expressed at mid-range levels when compared to the expression patterns in the white adipose tissue of *ad libitum* fed animals, unlike the liver. In contrast to our results, one previous study has reported rhythmic expression patterns of *Per1* and *Per2* in eWAT of restricted-fed animals, but perhaps the difference results from the manner in which the studies were conducted [20]. The restricted feeding regimen used in *Zvonic et al*. was less severe (12 hour food availability as compared to our 6 hour feeding window), fewer time points were used within a 24 hour period than were used in our study, and *Zvonic et al.* placed animals on restricted feeding for seven days instead of twelve to fourteen [20]. We have also shown that *Bmal1* peak expression occurs three hours earlier in eWAT than in the liver on restricted feeding. *Bmal1* expression levels are approximately three-fold higher in the eWAT of restricted-fed animals than in the eWAT of *ad libitum* fed animals, whereas restricted feeding results in a decrease of peak expression of *Bmal1* in the liver. The tissue specific expression patterns of core clock genes in restricted-fed animals raises the possibility that the regulation of their expression may be achieved through unknown mechanisms or may be the result of different entraining signals.

The observation that *Nocturnin* is induced by fasting in restricted fed mice is the first example of *Nocturnin's* acute induction in the mouse. In *Drosophila melanogaster* it has been shown that *curled*, the homolog of *Nocturnin*, is induced by starvation further supporting a role for *Nocturnin* in fasting [29]. Our studies revealed that *Nocturnin's* induction is tissue specific and feeding regimen specific, as it only occurs in the white adipose tissue, and not in the liver, under a restricted feeding paradigm. Several possibilities could explain the feeding regimen specific induction of *Nocturnin* in eWAT. Gene expression in the rat salivary gland entrains to a feeding schedule after a sympathectomy has been performed, indicating that sympathetic input to the tissue modulates food entrainment and perhaps inhibits food entrainment in this tissue [30]. *Nocturnin's* inducibility in white adipose tissue may be suppressed when the phase of the tissue is entrained to the light-dark cycle, and this suppression could be relieved when the tissue phase-shifts in response to a feeding schedule.

Another potential explanation for the restricted-feeding specific induction of *Nocturnin* in response to fasting may be found in the observation that there are different methods of energy utilization during the different stages of food deprivation. The early fasting stage is categorically centered on glycogenolysis, and after glycogen stores in the liver are depleted, fuel sources are produced by gluconeogenesis and by lipolysis of white adipose tissue [31]. The difference in metabolic states between mice receiving one 16 hr fast and that of mice receiving daily 18 hr fasts may account for the induction of *Nocturnin* seen in restricted fed mice.

Perhaps another reason for the feeding paradigm-specific induction of *Nocturnin* is the fact that lipolysis in response to an acute stress, such as that presented to the restricted fed mice, is robust, transitory, and predominantly mediated by catecholamines [32]. Our data show that *Nocturnin* is induced by a rise in cAMP levels, a central step in the well-studied hormone-induced lipolysis cascade. We show that *Nocturnin* expression increases in response to forskolin, to IBMX, and to the overexpression of PKA in NIH3T3 fibroblasts (Figure 5). A previous study reported that *Nocturnin* is not induced by forskolin in NIH3T3 fibroblasts [14]. This seeming contradiction is likely the result of the differing treatment of the cells with forskolin. The previous study treated NIH3T3 fibroblasts with forskolin for 15 minutes, whereas our present study harvested NIH3T3 fibroblasts treated with forskolin for 2, 4, or 6 hours (Figure 5C). Our data also show that *Nocturnin* is induced by forskolin in mature 3T3-L1 adipocytes, further supporting the finding that *Nocturnin* expression increases in response to a rise in cAMP. The finding that *Nocturnin* is induced by a fast in white adipose tissue of restricted fed mice is contrary to the original hypothesis that *Nocturnin* would be induced by feeding. The fact that *Nocturnin* is not induced by feeding in liver or in white adipose tissue, while it is induced by insulin in 3T3-L1 preadipocytes [15], further highlights the tissue specificity and developmental specificity of *Nocturnin's* inducibility, positioning it to regulate a diverse set of transcripts throughout the organism in response to a variety of stimuli.

Mammalian Nocturnin has been shown to exhibit deadenylase activity *in vitro*, suggesting that its function is to control mRNA stability or translatability. However, direct mRNA targets of Nocturnin's post-transcriptional regulation have yet to be identified. How this function contributes to the fasting response in eWAT is unclear but many interesting possibilities exist. Perhaps Nocturnin contributes to the post-transcriptional control of mRNAs involved in lipolysis or adipocyte signaling [33], [34], [35]. The recent study showing Nocturnin promotes adipogenesis by increasing the nuclear translocation of PPARγ suggests that Nocturnin may have other functions in addition to deadenylase activity [15]. The observation that Nocturnin plays a role in adipogenesis, exhibits a food entrainable circadian expression profile in the liver (Figure 3A), and contains a leucine zipper motif in the *Xenopus laevis* model is reminiscent of another leucine zipper containing protein, glucocorticoid induced leucine zipper (GILZ) [15], [36], [37]. Perhaps the functions of GILZ may provide new insights into other roles of Nocturnin. Future studies investigating the role that *Nocturnin* plays in white adipose tissue, and how that differs from the role *Nocturnin* plays in the liver, will add to our knowledge regarding the crosstalk between circadian clocks and metabolism.

Future studies of *Nocturnin* should also investigate different white adipose tissue depots, as it has been shown that qualitative differences exist between different depots. For example, human visceral white adipose tissue exhibits a stronger lipolytic response to catecholamines than subcutaneous adipose tissue, and the anti-lipolytic affect of insulin is stronger in subcutaneous adipose tissue than visceral adipose tissue [38], [39]. Also, the expression of leptin is higher in epididymal white adipose tissue than in subcutaneous adipose tissue [40]. These differences among depots of white adipose tissue may also extend to the expression patterns of core clock genes and of *Nocturnin*, as well as the regulatory mechanisms behind the patterns.

It is interesting to entertain the possibility that white adipose tissue could play a prominent role in food entrainment. Given its ability to dynamically respond to food or the lack thereof, its ability to integrate a variety of systemic signals both neural and humoral, and its function as an endocrine organ, it is well positioned to aid the adaptation of the animal to the timing of food availability. Our studies suggest a role for *Nocturnin* in the white adipose tissue's response to fasting in restricted fed mice, and highlight the usefulness of *Nocturnin* and white adipose tissue as tools with which to investigate the intersection of circadian timing and metabolism.

## Materials and Methods

### Animals

All experiments were conducted under the guidelines set forth by the Institutional Animal Care and Use Committee at the University of Virginia. For all experiments, C57BL/6 male mice aged 4–6 months were used. Mice were housed in 12∶12 LD with *ad libitum* feeding to establish whether *Nocturnin* is rhythmically expressed in white adipose tissue, and to compare that expression profile to the liver as a control. All studies thereafter were conducted in 12∶12 LD to keep the photic stimuli consistent with the experiment conducted in Figure 1A. For all timepoints, mice were euthanized via CO_2_ followed by cervical dislocation. For time points conducted during the dark hours, mice were euthanized in the dark, and tissues were collected in the light. Specifically, for studies testing the dependence of *Nocturnin's* expression on food, mice were housed in 12∶12 LD, with running wheels, and were fed standard chow *ad libitum* for 7 days. Then food was removed at ZT12 (onset of darkness), and mice were euthanized 25 hours later, at ZT13. Tissues were harvested, frozen in liquid nitrogen, and stored at -80°C until further use. For studies testing the induction of *Nocturnin* expression in response to feeding after a fast, mice were habituated for 7 days to 12∶12 LD, with running wheels, and with *ad libitum* access to standard chow. On the eighth day, food was removed at ZT12. On day nine, the cohort was split and one group remained without food while the other group received a meal at ZT2. All mice were euthanized at ZT4, tissues were harvested and frozen in liquid nitrogen, and stored at −80°C until further use. For the restricted feeding experiments, mice were habituated to 12∶12 LD, with running wheels, and with *ad libitum* access to standard chow for 7 days. At ZT9 on the seventh day, food was removed. Each day, from this day on, food was placed into food bowls in the bottom of the cages at ZT3 and was removed at ZT9. *Ad libitum* fed control mice had free access to food, but also received the food bowls at the same time and in the same manner as the restricted fed mice. Mice were on restricted feeding for 12-14 days before harvesting tissues every three hours. Tissues were frozen in liquid nitrogen and were stored at -80°C until further use. To test the affect the absence of the expected meal would have on *Nocturnin* expression, mice were habituated for 7 days in cages with running wheels placed in 12∶12 LD with *ad libitum* access to standard chow. They were placed on restricted feeding, as described above, for 12 days. On the 13th day, no food was given at ZT3. Tissues were harvested at ZT4 and at ZT7, frozen on liquid nitrogen, and stored at -80°C until further use.

### RNA isolation and cDNA Synthesis

Tissues were homogenized in Trizol (Invitrogen, Carlsbad, CA) using Lysing Matrix D (QBiogene, Carlsbad, CA) and the FastPrep homogenizer (QBiogene, Carlsbad, CA). RNA isolation was conducted according to the Trizol manufacturer's instructions. Total RNA was DNAse-treated (Sigma Aldrich, St. Louis, MO), extracted by acidic phenol chloroform extraction (Ambion, Austin, TX) and precipitated with ammonium acetate (Ambion, Austin, TX) and ethanol. 500 ng of total RNA was used to synthesize cDNA using the iScript cDNA Synthesis kit (BioRad, Hercules, CA), according to manufacturer's instructions.

### Real-time PCR

Real-time PCR was conducted with the Single Color MyiQ Detection System and iQ SYBR Green Supermix (BioRad, Hercules, CA). See Table S1 for a list of the primer sequences used. Data acquired from the restricted fed mice, *ad libitum* fed mice, and the fasted restricted fed mice were normalized to acidic ribosomal phosphoprotein PO (36B4). The data are graphed and reported as the mean fold change above 36B4 levels (±SEM). CircWave v1.4 software (courtesy of Dr. Roelof Hut, http://www.euclock.org/) was used to analyze the rhythmicity of gene expression patterns. p-values reported are the result of an f-test, user defined alpha  = 0.5. Graphs marked with ‡ represent data sets to which no curve could be fit, and all other graphs have a p-value ≤0.05. Forskolin induction data were normalized to *Cyclophilin B*. Points represent mean and error bars represent the standard error of the mean (±SEM). T-tests assuming equal variances were performed (alpha  = 0.05) and the two-way p-values were reported.

### Cell culture

NIH3T3 fibroblasts were cultured in high glucose-L-glutamine DMEM (Invitrogen, Carlsbad, CA) with 10% Fetal Bovine Serum-Premium Select (Atlanta Biologicals, Lawrenceville, GA) at 37°C with 5% CO_2_. For induction experiments in NIH3T3 fibroblasts, cells were seeded and grown to full confluence before adding IBMX (to a final concentration of 0.5 mM) or forskolin (to a final concentration of 10 µM). The vehicle used to dissolve both forskolin and IBMX was ethanol. Cells were harvested 0, 2, 4, or 6 hours post-treatment by washing the cells once with cold phosphate buffered saline, scraping and lysing with Trizol (Invitrogen, Carlsbad, CA), and storing the lysates at −80°C until further use. Transient transfections were conducted using FuGene6 (Roche Applied Sciences, Indianapolis, IN) and plasmids pCMV-PKA or dominant negative pCMV-KCREB (Clonetech, Mountain View, CA). Cells were collected approximately 24 hours after transfection in the same manner as described before. 3T3-L1 pre-adipocytes were cultured in high glucose-L-glutamine DMEM (Invitrogen, Carlsbad, CA) supplemented with 10% Fetal Bovine Serum-Premium Select (Atlanta Biologicals, Lawrenceville, GA) at 37°C with 5% CO_2_. Pre-adipocytes were differentiated by growing to confluence and adding Differentiation Media: DMEM-hg-L-glutamine, 10% FBS, 10 µg/mL insulin (Sigma Aldrich, St. Louis, MO), 1 µM Dexamethasone (Sigma Aldrich, St.Louis, MO), 0.5 mM 3-Isobutyl-1-methylxanthine (Sigma Aldrich, St.Louis, MO), and 1 µM Rosiglitazone (Cayman Chemicals, Ann Arbor, MI), set as Day 0. Forty-eight hours later, media was replaced with DMEM-hg-L-glutamine, 10%FBS, 10 µg/mL insulin, 1 µM Rosiglitazone. Every 48 hours media was changed with standard culture media (DMEM-hg-L-glutamine 10%FBS) and 10 µg/mL insulin. Differentiation was confirmed by visual observation of cell morphology and forskolin induction experiments were conducted on Day 10 post-differentiation. For induction studies, 3T3-L1 adipocytes were first serum starved by adding serum free DMEM-hg-L-glutamine for two hours. Then 10 µM forskolin (Sigma Aldrich, St. Louis, MO) or vehicle (ethanol) was added to the cells and cells were collected 0, 2, and 6 hours post-treatment. Samples were collected as above and frozen at −80°C until further use.

### Promoter analysis

Mouse (AC118601) and human (AC093602) *Nocturnin* sequences were aligned, and the 10 kb region upstream of the translation start site was identified. These sequences were analyzed with MatInspector. Putative sites reported here have 100% conservation of the core sequence and 75% conservation of the matrix sequence.

## Supporting Information

Table S1 Primer sequences for quantitative RT-PCR.
(DOC)
