# Supplementary material for: Nocturnin Expression Is Induced by Fasting in the White Adipose Tissue of Restricted Fed Mice
Source: PLoS One. 2011 Feb 10;6(2):e17051. doi: 10.1371/journal.pone.0017051 (PMC3037405; doi:10.1371/journal.pone.0017051)
Supplement: Table S1 — Primer sequences for quantitative RT-PCR. (DOC) [file pone.0017051.s001.doc]

**Table S1. Primer sequences used for** qPCR.

| Gene | Forward Primer | Reverse Primer | T(°C) |
| --- | --- | --- | --- |
| *36B4* | AGATTCGGGATATGCTGTTGGC | TCGGGTCCTAGACCAGTGTTC | 60 |
| *CyclophilinB* | GGTGGAGAGCACCAAGACAGA | GCCGGAGTCGACAATGATG | 60 |
| *Nocturnin* | TCATGCAGTGGAACATCCTC | TCAGGCACTTCCTCTCTTCC | 56 |
| *Bmal1* | AAGTGCAACAGGCCTTCAGT | GGTGGCCAGCTTTTCAAATA | 60 |
| *Period1* | CAACCCATCTACCAGTGGCT | CAGTCGAAGTTTGAGCTCCC | 59.5 |
| *Period2* | GCAGGTGAAGGCTAATGAGG | GCAAACATATCCGCGTTCTT | 58 |
| *Dbp1* | GGAACTGAAGCCTCAACCAATC | CTCCGGCTCCAGTACTTCTCA | 54 |
